# Batf is important for IL-4 expression in T follicular helper cells

**DOI:** 10.1038/ncomms8997

**Published:** 2015-08-17

**Authors:** Anupama Sahoo, Andrei Alekseev, Kentaro Tanaka, Lidiya Obertas, Beatrisa Lerman, Cara Haymaker, Karen Clise-Dwyer, John S. McMurray, Roza Nurieva

**Affiliations:** 1 Department of Immunology, MD Anderson Cancer Center, Houston, Texas 77030, USA; 2 CNMC, Center for Cancer and Immunology Research, Children's National Medical Center, Washington, District of Columbia 20010, USA; 3 Department of Melanoma Medical Oncology, MD Anderson Cancer Center, Houston, Texas 77030, USA; 4 Division of Cancer Medicine, Department of Stem Cell Transplantation and Cellular Therapy, MD Anderson Cancer Center, Houston, Texas 77030, USA; 5 Division of Cancer Medicine, Department of Experimental Therapeutics, MD Anderson Cancer Center, Houston, Texas 77030, USA

## Abstract

Apart from T helper (Th)-2 cells, T follicular helper (Tfh) cells are a major class of IL-4-producing T cells, required for regulation of type 2 humoral immunity; however, transcriptional control of IL-4 production in Tfh cells remains mainly unknown. Here, we show that the basic leucine zipper transcription factor ATF-like, Batf is important for IL-4 expression in Tfh cells rather than in canonical Th2 cells. Functionally, Batf in cooperation with interferon regulatory factor (IRF) 4 along with Stat3 and Stat6 trigger IL-4 production in Tfh cells by directly binding to and activation of the CNS2 region in the IL-4 locus. In addition, Batf-to-c-Maf signalling is an important determinant of IL-4 expression in Tfh cells. Batf deficiency impairs the generation of IL-4-producing Tfh cells that results in protection against allergic asthma. Our results thus indicate a positive role of Batf in promoting the generation of pro-allergic IL-4-producing Tfh cells.

## Abstract (web summary)

T follicular helper cells are a distinct subtype of CD4 T helper cells, which contributes to the regulation of type 2 humoral immunity by producing IL-4. Here, the authors identify Batf as an important transcription factor regulating IL-4 expression in T follicular helper cells but not in Th2 cells.

## Graphical abstract

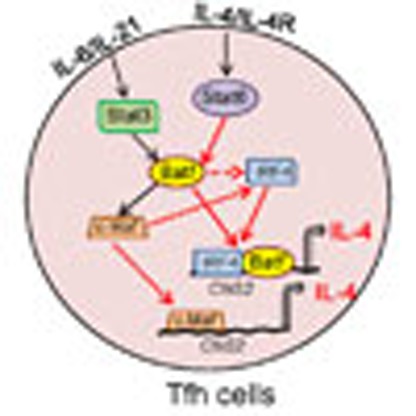

Interleukin-4 (IL-4) was originally identified as a B cell-stimulating factor critical for class-switch recombination of B cells to IgG1- and IgE-producing cells and is strongly implicated in atopic and allergic diseases^1^. In addition to the T helper (Th)-2 cell subset, which is known to be the main source of IL-4, recent findings have identified T follicular helper (Tfh) cells as an alternative source of IL-4 to regulate type 2 humoral immune responses^2^,^3^.

Cytokine gene expression in various Th subsets is usually accompanied by changes in chromatin structure and the accessibility of *trans*-acting factors to their binding sites in proximal gene promoters as well as distal *cis*-regulatory DNA elements^4^,^5^. The Th2-specific master transcriptional factors Stat6 and Gata3 have been shown to regulate Th2 differentiation and IL-4 expression^6^,^7^. In addition, nuclear factor of activated T (NFAT) cells, activating protein (AP)-1, JunB, c-Maf and interferon regulatory factor 4 (IRF4) are important for IL-4 production^8^,^9^,^10^. In contrast to Th2 cells, regulation of IL-4 expression in Tfh cells exclusively depends on the 3′conserved non-coding sequence 2 (CNS2) region in the IL-4 locus^11^,^12^, however, exact transcriptional regulation of IL-4 expression in Tfh cells is poorly understood.

The basic leucine zipper transcription factor ATF-like (Batf) belongs to the AP-1 family and displays unique positive transcriptional activities in T, B and dendritic cells^13^,^14^,^15^,^16^. Batf in cooperation with IRF4 plays a critical role in Th17 and Th9 cell differentiation by binding to the *Il17*, *Il21*, *Il22* and *Il9* gene promoters and controlling their expression^17^,^18^. Batf also controls the Tfh cell subset by directly binding to and regulating the Bcl-6 and c-Maf genes that are important for the Tfh cell lineage commitment^15^. In addition, *Batf*-deficient mice show diminished capacity to produce Th2-related cytokines and consequently to promote allergic inflammation^17^,^19^, suggesting the role of Batf in regulation of Th2 programming; however, the molecular mechanism whereby Batf controls Th2-related factors is still unknown. Moreover, the efficacy of Batf to control IL-4 cytokine in the Tfh cells remains to be investigated.

In the present study, we show that Batf contributes to the IL-4 expression in Tfh cells rather than in Th2 cells and to their pro-allergic function. We demonstrate that the Batf/IRF4 complex along with Stat3 and Stat6 aids IL-4 production in Tfh cells by direct binding and activation of the CNS2 region in the IL-4 locus. Moreover, IL-4–Stat6 signalling contributes to the Batf induction in Tfh cells. Collectively, our results show that Batf plays an important role in the generation of IL-4-expressing Tfh cells.

## Results

### Batf is essential for IL-4 expression in Tfh cells

*Batf*, a transcription factor of the AP-1/Jun family is known to play a crucial role in the development and function of multiple Th subsets^16^,^20^. Recent studies have emphasized on the crucial role of Batf in the development of allergic inflammation and possibly in controlling the Th2 developmental programme^17^,^19^. We subjected both wild-type (WT) and *Batf* knockout (KO) mice to either primary immunization with ovalbumin (Ova) in aluminium hydroxide (Alum) or asthma as described in the Methods section. Consistently^19^, our results from *in vivo* models show that Batf deficiency in mice leads to a global defect in Th2-related cytokines (Supplementary Fig. 1a–c). To further assess whether the decreased Th2 responses in *Batf* KO mice are T-cell intrinsic, we transferred naive WT and *Batf* KO CD4^+^ cells into *TCR-β* KO mice followed by Ova in Alum immunization. Similar to above results, mice reconstituted with *Batf* KO cells showed decreased expression of Th2 cytokines and IL-4-dependent IgGs compared with mice that received WT cells (Supplementary Fig. 1d,e) suggesting that Batf function in T cells is required for expression of Th2 cytokines *in vivo*.

On the basis of the T-cell-intrinsic role of Batf in regulation of Th2 signature cytokines, we next examined the mechanism whereby Batf regulates Th2 responses. Surprisingly, analysis of naive WT and *Batf* KO CD4^+^ T cells activated under Th2 polarizing conditions *in vitro* revealed unaltered *Il4* mRNA expression in *Batf* KO Th2 cells compared with WT cells (Supplementary Fig. 2a), while the expression of other Th2 signature cytokines like *Il5*, *Il13* and the master Th2 transcription factor *Gata3* was decreased. Chromatin immunoprecipitation (ChIP) analysis further revealed enhanced recruitment of Batf to the Gata3 promoter in WT Th2 cells (Supplementary Fig. 2b), while the recruitment of active histone proteins, histone H3 acetylation (AcH3) and trimethyl histone H3 lysine 4 (H3k4) was decreased at the Gata3 promoter in the absence of Batf (Supplementary Fig. 2c) suggesting Batf selectivity in the regulation of Th2 programming.

**Figure 1:**
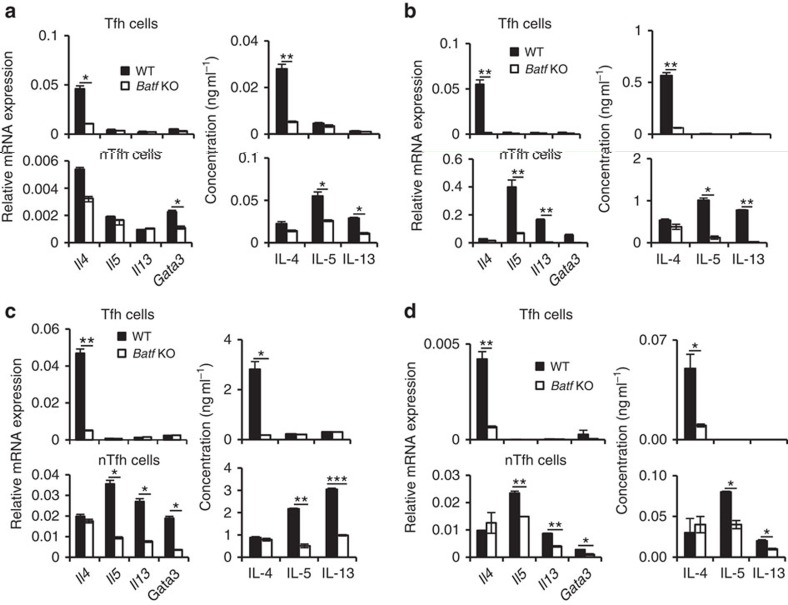
Batf-dependent regulation of IL-4 in Tfh cells. (**a**) Male WT and *Batf* KO mice (6–8 weeks old, *n*=5 per group) were injected i.p. with 0.2 ml saline containing 100 μg Ova in Alum. Seven days later, splenocytes from immunized mice were isolated and stimulated with Ova (100 μg ml^−1^) for 72 h *ex vivo*. CD4^+^CD44^hi^CXCR5^hi^PD1^hi^ (Tfh) and CD4^+^CD44^hi^CXCR5^−^ (nTfh) cells were then sorted as described in the Methods section and expression of the indicated cytokines was analysed by qRT–PCR and ELISA. (**b**) FACS-sorted naive CD4^+^ T cells from male WT and *Batf* KO (6–8 weeks old) mice were intravenously transferred into *TCR-β* KO (6–8 weeks old, 10 million cells per mouse, *n*=4 per group) mice followed by i.p. injection with Ova in Alum. Seven days later splenocytes were restimulated with Ova *ex vivo* and Tfh and nTfh cells from the spleen were sorted and effector cytokine levels were analysed as in (**a**). (**c**) Naive CD4^+^ T cells from male WT OTII and *Batf* KO OTII (6–8 weeks old) mice were transferred to male B6.SJL (CD45.1^+^) mice (6–8 weeks old, 10 million cells per mouse; *n*=5 per group) followed by immunization with Ova in Alum 24 h post transfer. Seven days post immunization splenocytes from immunized mice were isolated and stimulated with Ova (100 μg ml^−1^) for 72 h *ex vivo.* Tfh and nTfh cells were sorted and analysed as in (**a**). (**d**) Mixed bone marrow chimeric mice (*n*=10) were generated by co-transfer of bone marrow cells from male WT (CD45.1^+^) and *Batf* KO (CD45.2^+^) mice into sublethally irradiated male *Rag1* KO (6–8 weeks old) mice. Eight weeks later, mice were immunized with Ova in Alum and Tfh and non-Tfh cells from the spleen of immunized mice were sorted and analysed as in (**a**). The qRT–PCR data shown in (**a**–**d**) were normalized by the expression of a reference gene *Actb*. Results shown are mean±s.e.m. and representative of at least three independent experiments. *P* values: *<0.05 and **<0.01. Student's *t*-test was performed to detect between-group differences.

According to a recent study, Tfh cells serve as a alternative source of IL-4 in a helminth infection model^2^. Since Batf deficiency did not affect IL-4 expression in Th2 cells *in vitro* (Supplementary Fig. 2a), the dramatic decrease in IL-4 expression *in vivo* in *Batf* KO mice could be potentially attributed to Tfh cells^2^,^11^. To address this possibility, we stimulated splenocytes from Ova-immunized WT and *Batf* KO mice with Ova *ex vivo* for 3 days and sorted and analysed CD4^+^CD44^hi^CXCR5^hi^PD1^hi^ (Tfh) and CD4^+^CD44^hi^CXCR5^−^ (nTfh) cells as described in the Methods section (Supplementary Fig. 3; Fig. 1a). Consistent with *in vitro*-differentiated Th2 cells, Batf deficiency in nTfh cells led to decreased expression of IL-5 and IL-13 as well as *Gata3*, but not IL-4 (Fig. 1a). However, the decrease in IL-4 level was prominent in *Batf* KO Tfh cells both at mRNA and protein levels (Fig. 1a). To further demonstrate whether this profound defect in IL-4 production by Batf-deficient Tfh cells is T-cell intrinsic, we sorted and analysed Tfh and nTfh cells from *TCR-β* KO mice reconstituted with naive WT and *Batf* KO CD4^+^ T cells and subjected to Ova in Alum immunization (Fig. 1b). Tfh cells from mice reconstituted with Batf-deficient CD4^+^ T cells showed a consistent defect in IL-4 expression compared with Tfh cells from mice, which received WT CD4^+^ T cells, while IL-4 level remained unaltered in WT and *Batf* KO nTfh cells (Fig. 1b). To confirm that the acquired Tfh cell phenotype was truly antigen specific, we adoptively transferred naive WT and *Batf* KO Ova transgenic (OT) II cells into B6.SJL (CD45.1^+^) mice and immunized them with Ova in Alum. Seven days post immunization donor WT and *Batf* KO Tfh and nTfh cells were sorted from the spleen of these mice and IL-4, IL-5 and IL-13 levels were analysed by quantitative reverse-transcription PCR (qRT–PCR) and enzyme-linked immunosorbent assay (ELISA) (Fig. 1c). Similar to our above observations, *Batf* KO OTII Tfh cells showed lower IL-4 level compared with WT cells, while IL-4 level was comparable between WT and KO OTII nTfh cells (Fig. 1c).

We further generated mixed bone marrow chimeras by transferring a mixture of congenic CD45.1^+^ WT and CD45.2^+^ Batf-deficient bone marrow cells into sublethally irradiated *Rag1* KO mice. Eight weeks after reconstitution, we immunized mice with Ova protein in Alum. Similarly, we observed significant defect in IL-4 expression only by *Batf* KO Tfh cells compared with WT Tfh cells (Fig. 1d). Thus, our results indicate towards an important intrinsic role of Batf in regulating IL-4 expression specifically in Tfh cells and further endorse the pro-allergic function for IL-4-producing Tfh cells.

### Batf contributes to pro-allergic function of Tfh cells

**Figure 2:**
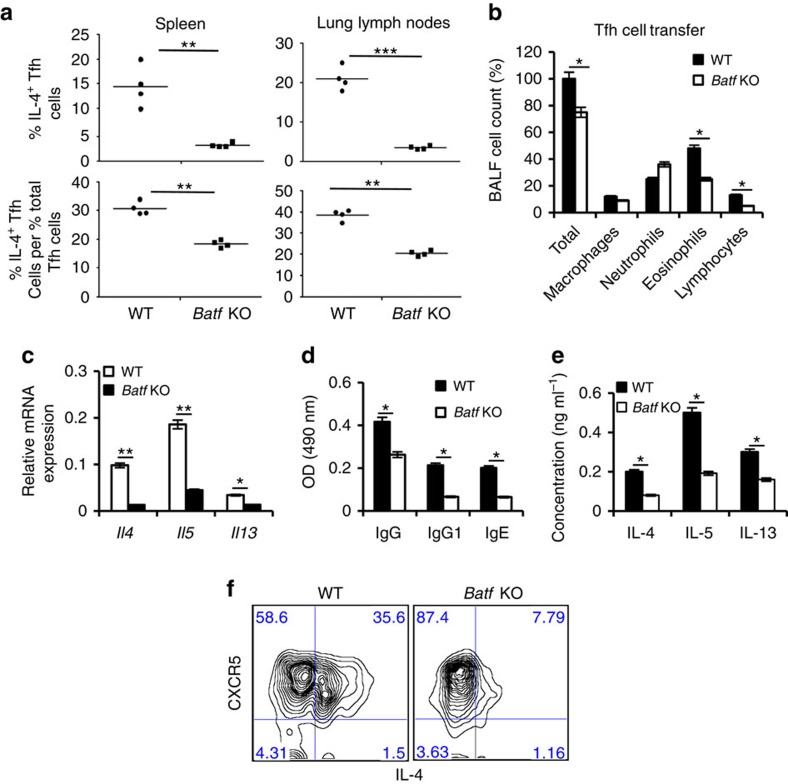
Batf contributes to pro-allergic function of IL-4-expressing Tfh cells. (**a**) Male WT and *Batf* KO mice (6–8 weeks old, *n*=5 per group) were injected i.p. with 0.2 ml saline containing 100 μg Ova in Alum at 2 weeks interval. On day 14, mice were intranasally challenged with Ova followed by three more challenges at days 26, 27 and 28. Twenty-four hours after the last challenge mice were killed. Percentage of IL-4-expressing CD4^+^CD44^hi^CXCR5^hi^PD1^hi^ (Tfh) cells and ratio of percentage of IL-4-expressing Tfh cells to percentage of total Tfh cells from spleen and lung lymph nodes of WT and *Batf* KO asthmatic mice were analysed by flow cytometry. (**b**–**f**) Bone marrow chimera mice were generated and subjected to asthma as described in the Methods section. WT (CD45.1^+^) and *Batf* KO (CD45.2^+^) Tfh cells from the asthmatic mice were sorted and transferred into male C57BL/6 (CD45.2^+^) or B6.SJL (CD45.1^+^; 6–8 weeks old, *n*=4) mice, respectively. Twenty-four hours later the mice were challenged with Ova intranasally for 5 days and analysed. (**b**) BALF was analysed to measure airway infiltrating cells. (**c**) Expression of indicated cytokines in the lung was analysed by qRT–PCR analysis. Data were normalized to beta-actin gene. (**d**) Levels of Ova-specific IgGs in the serum were analysed by ELISA. (**e**) Spleen cells from the recipient mice were restimulated with Ova for 72 h and effector Th2 cytokines were analysed by ELISA. (**f**) Lung and lung lymph node cells were stained with Pacific blue-labelled CD45.1 mAb, PerCP-labelled anti-CD4 mAb, FITC-labelled anti-CD44 mAb and biotinylated anti-CXCR5 mAb, followed by APC-labelled streptavidin (BD Biosciences). Donor WT (CD45.1^+^) and *Batf* KO (CD45.2^+^) CD4^+^CD44^hi^ cells were assessed for CXRCR5 and IL-4 expression after restimulation with Ova for 24 h. Results shown are mean±s.e.m. and representative of at least two independent experiments. *P* values: *<0.05, **<0.01 and ***<0.001. Student's *t*-test was performed to detect between-group differences.

The implication of Th2 cells in the pathogenesis of allergic asthma is well defined^21^. Interestingly, lower asthmatic symptoms observed in *Batf* KO mice correlated with a significantly lower percentage of IL-4-producing Tfh cells compared with WT mice (Fig. 2a) indicating that Tfh cells are dependent on Batf for IL-4 expression during allergic inflammation. To clarify whether Batf-mediated IL-4 expression in Tfh cells is involved in triggering asthmatic symptoms, we used an adoptive transfer model of asthma. We generated mixed bone marrow chimeras by transferring a mixture of congenic CD45.1^+^ WT and CD45.2^+^ Batf-deficient bone marrow cells into sublethally irradiated *Rag1* KO mice. Eight weeks after reconstitution, we subjected the mice to asthma, sorted out WT (CD45.1^+^CD4^+^CD44^hi^CXCR5^hi^PD1^hi^) and *Batf* KO (CD45.2^+^ CD4^+^CD44^hi^CXCR5^hi^PD1^hi^) Tfh cells and intravenously transferred them into congenic C57BL6 (CD45.2^+^) or B6.SJL (CD45.1^+^) mice, respectively followed by intranasal challenge with Ova for 5 days. Transfer of *Batf* KO Tfh cells resulted in decreased numbers of total infiltrated cells, eosinophils and lymphocytes in recipient mice (Fig. 2b) along with decreased Th2 cytokines IL-4, IL-5 and IL-13 in their lungs (Fig. 2c) compared with mice that received WT Tfh cells suggesting that Batf-dependent IL-4 expression in Tfh cells is essential for the development of asthmatic symptoms. Consistently, the IL-4-dependent immunoglobulins in the sera were significantly diminished (Fig. 2d). We also observed a decrease in the IL-4, IL-5 and IL-13 cytokines on Ova restimulation of splenocytes in mice that received Batf-deficient Tfh cells (Fig. 2e), which could be due to defective IL-4 production by the transferred Batf-deficient Tfh cells, thus protecting recipient mice from disease establishment. On the other hand, mice that received WT Tfh cells showed increased endogenous Th2 responses, thus triggering allergic inflammation. Importantly, the ability of donor Tfh cells to maintain their phenotype after transfer further supported the role of Tfh cells in allergic inflammation (Fig. 2f). To further confirm whether allergic inflammation is triggered by antigen-specific Tfh cells, we sorted donor Tfh cells from asthmatic mice that received either naive WT OTII or *Batf* KO OTII CD4^+^ T cells and transferred them into B6.SJL (CD45.1^+^) mice (10^5^ cells per mouse) followed by intranasal challenge with Ova for 5 days post transfer. Consistent with above results, mice that received Ova-specific *Batf* KO Tfh cells revealed decreased numbers of total infiltrated cells, eosinophils and lymphocytes (Supplementary Fig. 4a) along with decreased expression of Th2 cytokines IL-4, IL-5 and IL-13 by Ova-restimulated splenocytes (Supplementary Fig. 4b) compared with mice that received WT Tfh cells. The IL-4-dependent immunoglobulins in the sera of mice that received *Batf* KO OTII T cells were also significantly diminished (Supplementary Fig. 4c). Altogether, these results suggest that the IL-4-producing Tfh cells are involved in the pathogenicity of allergic asthma and Batf is important in controlling the IL-4 level in these cells.

### Batf/IRF4 complex regulates IL-4 production in Tfh cells

**Figure 3:**
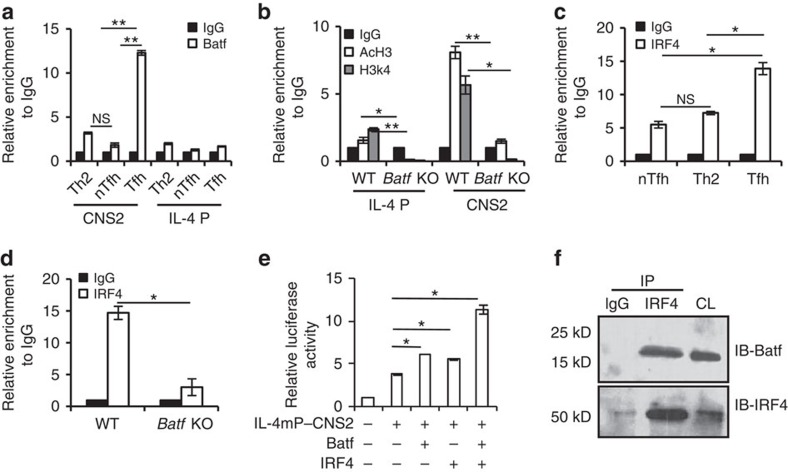
Batf and IRF4 regulate IL-4 expression in Tfh cells through the CNS2 locus. (**a**) Chromatin immunoprecipitation (ChIP) analysis of Batf binding at the IL-4 locus in *in vitro*-differentiated Th2 cells and nTfh and Tfh cells isolated from Ova-immunized male WT(6–8 weeks old) mice. (**b**) ChIP analysis of active histone proteins, H3 acetylation (AcH3) and trimethyl histone H3 lysine 4 (H3k4) at the IL-4 locus in Tfh cells isolated from Ova-immunized male WT and *Batf* KO (6–8 weeks old) mice. (**c**) ChIP analysis of IRF4 binding at the CNS2 locus in *in vitro*-differentiated Th2 cells and nTfh and Tfh cells isolated from Ova-immunized male WT (6–8 weeks old) mice. (**d**) ChIP analysis of IRF4 binding in Tfh cells isolated from Ova-immunized male WT and *Batf* KO (6–8 weeks old) mice. (**e**) Luciferase assay in EL-4 cells transfected with IL-4 minimal promoter containing CNS2 luciferase vector (IL-4mP–CNS2) along with vectors containing the indicated factors. Relative luciferase units are expressed as a fold difference to the control (pGL3) value. (**f**) CD4^+^CD44^hi^CXCR5^hi^PD1^hi^ (Tfh) were sorted from the spleen of Ova-immunized male WT (6–8 weeks old) mice as described in the Methods section. The lysates were subjected to IP using anti-IRF4 antibody. The blot was probed with anti-Batf antibody (upper panel) and reprobed with anti-IRF4 antibody (lower panel) as a control. The WCL was immunoblotted with anti-Batf or anti-IRF4 antibody for checking antibody efficacy. Results shown are mean±s.e.m. and representative of at least three independent experiments. *P* values: *<0.05 and **<0.01. Student's *t*-test was performed to detect between-group differences.

IL-4 expression is governed by a set of coordinated changes occurring at the IL-4 locus including chromatin remodelling, epigenetic changes and accessibility to various transcription factors^22^. Although it has been reported that the 3′ distal enhancer region CNS2 regulates IL-4 expression in both Th2 and Tfh cells^12^,^23^,^24^ in contrast to Th2 cells, IL-4 production in Tfh cells is solely CNS2 dependent^12^. To assess whether Batf controls IL-4 expression in Tfh cells through the CNS2 locus, we performed ChIP analysis in Tfh, nTfh and Th2 cells. There was no significant binding of Batf to the CNS2 region in either Th2 or nTfh cells. However, compared with Th2 and nTfh cells, enriched binding of Batf was observed in Tfh cells to the CNS2 region (Fig. 3a), but not to the IL-4 promoter. Further analysis revealed more pronounced enrichment of active histone proteins, AcH3 and H3k4 in the CNS2 region in Tfh cells compared with the IL-4 promoter (Fig. 3b). In addition, the occurrence of active histone proteins was markedly decreased in the absence of Batf indicating that Batf regulates IL-4 expression in Tfh cells through direct binding to the CNS2 region (Fig. 3b).

Transcription factors usually partner with other transcription factors or chromatin-modifying complexes to regulate gene expression^4^. Batf is known to functionally cooperate with IRF4 to regulate expression of genes encoding IL-17, IL-21, IL-22, IL-23R and IL-10 in Th17 cells and IL-9 in Th9 cells^16^,^17^,^20^. We also detected increased IRF4 binding to the CNS2 locus in Tfh cells compared with Th2 and nTfh cells (Fig. 3c) and this binding in Tfh cells was compromised in the absence of Batf (Fig. 3d) indicating a possible cooperation between Batf and IRF4 in the regulation of IL-4 expression in Tfh cells. By using dual-luciferase reporter assays, we found that while Batf and IRF4 alone could transactivate the IL-4 minimal promoter (85 bp to +10 bp) containing CNS2 region (IL-4mP–CNS2), overexpressing both of them further enhanced IL-4mP–CNS2 activity (Fig. 3e). Moreover, immunoprecipitation experiments performed in *in vivo*-generated Tfh cells revealed a direct interaction between endogenous Batf and IRF4 proteins (Fig. 3f, Supplementary Fig. 7), thus suggesting that Batf and IRF4 both physically and functionally cooperate and contribute to IL-4 expression in Tfh cells.

### Batf-c-Maf axis contributes to IL-4 expression in Tfh cells

In Tfh cells, Batf directly controls the expression of transcriptional factors Bcl-6 and c-Maf, both of which are critical regulators of Tfh cell development^15^. c-Maf regulates IL-21 production, which in turn regulates the expansion of Tfh cells; since, loss of c-Maf resulted in defective IL-21 production and fewer number of Tfh cells^25^. Besides regulation of IL-21 expression in Tfh cells, c-Maf serves as an IL-4-inducing factor in Th2 cells^8^,^26^; however, to date, it is not determined whether c-Maf contributes to IL-4 expression in Tfh cells. Consistent with the published finding, Batf deficiency leads to abolished c-Maf expression in both Tfh and nTfh cells (Supplementary Fig. 5a). Correspondingly, ChIP assay showed decreased recruitment of active histone proteins to the c-Maf locus in both Batf-deficient Tfh and nTfh cells (Supplementary Fig. 5b). However, we observed more pronounced enhancement of c-Maf binding to the CNS2 region in Tfh cells compared with nTfh cells (Supplementary Fig. 5c). Luciferase assays also showed transactivation of the IL-4mP–CNS2 reporter on c-Maf overexpression (Supplementary Fig. 5d). Thus, our results indicate that Batf-to-c-Maf signalling is an additional important determinant that controls IL-4 expression in Tfh cells.

### Stat3 and Stat6 contribute to IL-4 regulation in Tfh cells

**Figure 4:**
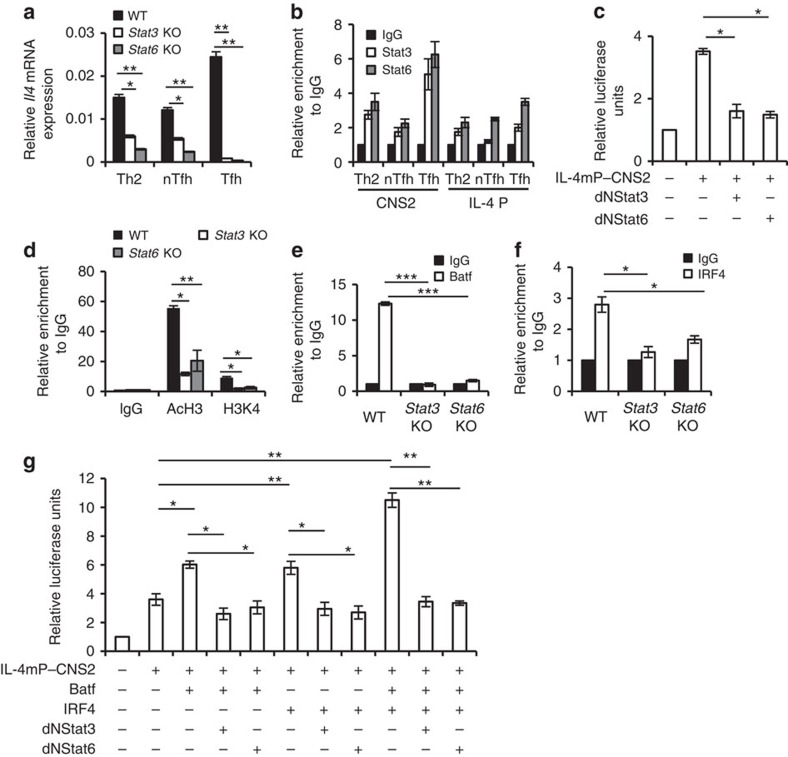
Batf cooperates with Stat3 and Stat6 to regulate IL-4 expression in Tfh cells. (**a**) *Il4* mRNA expression in *in vitro*-differentiated Th2 cells and nTfh and Tfh cells isolated from Ova-immunized male WT, *Stat3* KO and *Stat6* KO (6–8 weeks old) mice. The data shown were normalized by the expression of a reference gene *Actb*. (**b**) ChIP analysis of Stat3 and Stat6 binding to IL-4 locus in *in vitro*-differentiated Th2 cells and nTfh and Tfh cells isolated from Ova-immunized male WT, *Stat3* KO *and Stat6* KO (6–8 weeks old) mice. (**c**) Luciferase assay in EL-4 cells transfected with IL-4 minimal promoter containing CNS2 luciferase vector (IL-4mP–CNS2) in the presence of either double-negative mutant Stat3(dNStat3) or dNStat6. (**d**) ChIP analysis of active histone proteins (AcH3 and H3k4) at CNS2 region in Tfh cells isolated from Ova-immunized male WT, *Stat3* KO and *Stat6* KO (6–8 weeks old) mice. (**e**,**f**) ChIP analysis of Batf binding (**e**) and IRF4 binding (**f**) at CNS2 region in Tfh cells isolated from Ova-immunized male WT, *Stat3* KO and *Stat6* KO (6–8 weeks old) mice. (**g**) Luciferase assay in EL-4 cells transfected with IL-4mP–CNS2 luciferase vector along with vectors containing the indicated factors. Relative luciferase units are expressed as a fold difference to the control (pGL3) value. Results shown are mean±s.e.m. and representative of at least three independent experiments. *P* values: *<0.05, **<0.01 and ***<0.001. Student's *t*-test was performed to detect between-group differences.

Both Stat3 and Stat6 are known to play crucial roles in the regulation of IL-4 expression^6^,^27^. Our results also showed that IL-4 expression in Tfh, nTfh and Th2 cells depends on both Stat3 and Stat6 (Fig. 4a). In addition, we observed that both Stat3 and Stat6 showed pronounced binding to the CNS2 region in Tfh cells compared with nTfh and Th2 cells, but not with the IL-4 promoter (Fig. 4b), and inhibition of either Stat3 or Stat6 activity resulted in decreased IL-4mP–CNS2 luciferase activity (Fig. 4c). Furthermore, we detected that recruitment of AcH3 and H3k4 proteins to the CNS2 region was decreased in the absence of either Stat3 or Stat6 (Fig. 4d) suggesting that IL-4 expression in Tfh cells is influenced by both Stat3 and Stat6.

Next, we assessed whether Stat3 or Stat6 control the function of Batf–IRF4 towards regulation of IL-4 expression in Tfh cells. Interestingly, ChIP analysis revealed that binding of both Batf and IRF4 was significantly compromised in Tfh cells in the absence of either Stat3 or Stat6 (Fig. 4e,f). Moreover, dual-luciferase reporter assay showed that Batf- and IRF4-mediated transactivation of IL-4mP–CNS2 locus was completely abolished on dysfunction of either Stat3 or Stat6 (Fig. 4g) indicating that Stat3 and Stat6 contribute to the binding of Batf–IRF4 complex to CNS2 region in IL-4 locus and thereafter to IL-4 expression in Tfh cells.

### Batf and c-Maf contribute to IRF4 expression in Tfh cells

**Figure 5:**
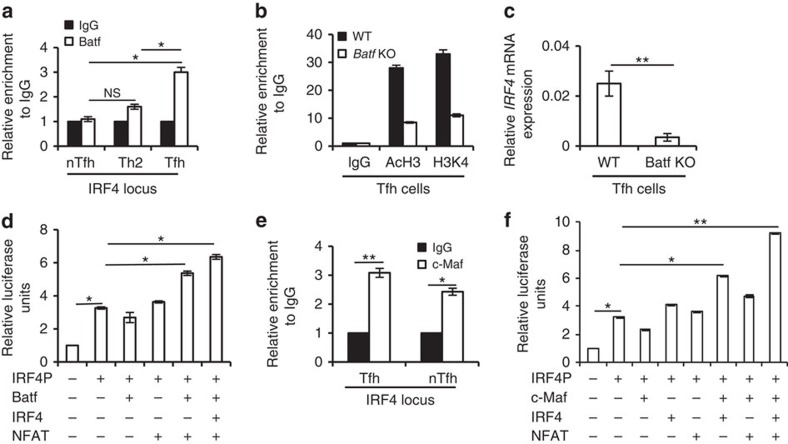
Batf and c-Maf regulate IRF4 expression. (**a**) ChIP analysis of Batf binding in IRF4 locus in *in vitro*-differentiated Th2 cells and nTfh and Tfh cells isolated from Ova-immunized male WT (6–8 weeks old) mice. (**b**) ChIP analysis of active histone proteins (AcH3 and H3k4) at the IRF4 locus in Tfh cells isolated from Ova-immunized male WT and *Batf* KO (6–8 weeks old) mice. (**c**) *IRF4* mRNA expression in Tfh cells isolated from Ova-immunized male WT and *Batf* KO mice. Data were normalized to beta-actin gene. (**d**) Luciferase assay in EL-4 cells transfected with IRF4 promoter containing luciferase vector along with expression plasmids of the indicated factors. Relative luciferase units are expressed as a fold difference to the control (pGL3) value. (**e**) ChIP analysis of c-Maf binding to the IRF4 locus in Tfh and nTfh cells isolated from Ova-immunized male WT (6–8 weeks old) mice. (**f**) Luciferase assay in EL-4 cells transfected with IRF4 promoter containing luciferase vector along with expression plasmids of the indicated factors. Relative luciferase units are expressed as a fold difference to the control (pGL3) value. Results shown are mean±s.e.m. and representative of at least three independent experiments. *P* values: *<0.05 and **<0.01. Student's *t*-test was performed to detect between-group differences.

Interestingly, our further experiments revealed that Batf also binds to the IRF4 locus in Tfh cells compared with Th2 and nTfh cells (Fig. 5a). In addition, Batf-deficient Tfh cells showed decreased active histone modifications at the IRF4 locus and IRF4 mRNA expression compared with WT Tfh cells indicating Batf-dependent regulation of IRF4 in Tfh cells (Fig. 5b,c). Luciferase assays in EL-4 cells did not show transactivation of the IRF4 promoter on Batf overexpression suggesting that Batf could cooperate with other transcriptional factors towards IRF4 induction. It has been reported that NFAT activity is necessary for IRF4 expression^28^. In addition, IRF4 itself could induce its own expression^29^. However, overexpression of either IRF4 or NFAT alone did not transactivate the IRF4 promoter (Fig. 5d,f). In addition, IRF4 transactivation does not require cooperation between Batf and IRF4 (Fig. 5d). However, co-transfection of Batf and NFAT was sufficient to induce IRF4 expression (Fig. 5d). Thus, the above results indicated a novel function of Batf in the regulation of IRF4 expression in Tfh cells.

We also detected more profound binding of c-Maf to the IRF4 locus in Tfh cells compared with nTfh cells (Fig. 5e). Similarly to Batf, c-Maf alone could not transactivate IRF4 promoter (Fig. 5f). It has been reported that c-Maf in cooperation with IRF4 and NFAT induces IL-4 expression^10^. Our analysis revealed that co-expression of c-Maf and IRF4 significantly triggered IRF4 promoter transactivation (Fig. 5f). Moreover, overexpression of NFAT in addition to c-Maf and IRF4 further enhanced IRF4 promoter activity (Fig. 5d). Thus, c-Maf–IRF4 complex along with NFAT could induce IRF4 expression in Tfh cells.

### Regulation of Batf expression

Although it is known that Batf is highly expressed in Tfh cells on IL-6–Stat3 signalling, analysis of Th0, Th1, Th2, Th17 and Treg cells differentiated *in vitro* and in *in vivo*-generated Tfh and nTfh cells revealed significantly enhanced *Batf* expression also in Th2 cells (Supplementary Fig. 6a). Concurrent with the mRNA levels, enrichment of active histone proteins (AcH3 and H3k4) was observed at the *Batf* locus in both Th2 and Tfh cells (Supplementary Fig. 6b) suggesting that Batf expression in Tfh cells could have a regulation mechanism similar to Th2 cells.

**Figure 6:**
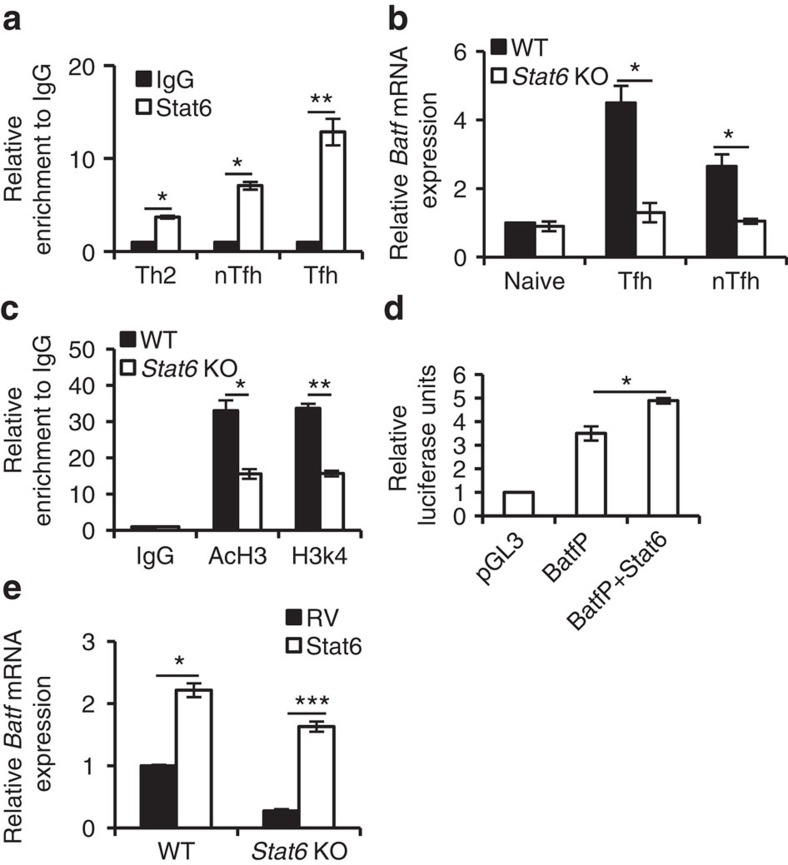
Expression of Batf in Tfh cells. (**a**) ChIP analysis of Stat6 binding to Batf locus in *in vitro*-differentiated Th2 cells and in nTfh and Tfh cells isolated from Ova-immunized male WT (6–8 weeks old) mice. (**b**) *Batf* mRNA expression in naive, Tfh and nTfh cells isolated from Ova-immunized male WT and *Stat6* KO (6–8 weeks old) mice. (**c**) ChIP analysis of active histone proteins (AcH3 and H3k4) at Batf locus in Tfh cells isolated from Ova-immunized male WT and *Stat6* KO (6–8 weeks old) mice. (**d**) Luciferase assay in EL-4 cells transfected with Batf promoter (BatfP) containing luciferase vector along with vector containing Stat6. Relative luciferase units are expressed as a fold difference to the control (pGL3) value. (**e**) *Batf* expression in naive WT and *Stat6* KO CD4^+^ T lymphocytes infected with bicistronic retrovirus expressing STAT6. The data shown in (**b**) and (**e**) were normalized by the expression of a reference gene *Actb*. Results shown are mean±s.e.m. and representative of at least three independent experiments. *P* values: *<0.05, **<0.01 and ***<0.001. Student's *t*-test was performed to detect between-group differences.

To assess this possibility, we analysed the binding of Th2-related transcription factors including Stat6, Gata3, IRF4, NFATc1, JunB and c-Maf to *Batf* locus in Tfh cells. Interestingly, among these factors, ChIP analysis revealed significant binding of Stat6 to the *Batf* locus (Fig. 6a). Moreover, Stat6 binding to the Batf locus was more pronounced in Tfh cells compared with Th2 and nTfh cells (Fig. 6a). In addition, we also found that *Batf* mRNA expression was augmented in a time-dependent manner on IL-4 treatment (Supplementary Fig. 6c) emphasizing on the role of IL-4/Stat6 in the regulation of *Batf* expression in Tfh cells. In support, analysis of Stat6-deficient Tfh showed a significant decrease in *Batf* mRNA expression (Fig. 6b) as well as active histone modifications (AcH3 and H3k4) in *Batf* locus compared with WT Tfh cells (Fig. 6c). Moreover, dual-luciferase reporter assays in EL-4 cells revealed that Stat6 could directly bind and enhance the *Batf* promoter activity (Fig. 6d). In addition, overexpression experiments showed that Stat6 not only can increase *Batf* expression in WT T cells but also can rescue *Batf* expression in Stat6-deficient cells (Fig. 6e). Together, these findings suggest that in addition to IL-6/STAT3 signalling, IL-4–Stat6 axis could contribute to triggering Batf expression in Tfh cells.

## Discussion

Tfh cells have recently emerged as the major producers of IL-4 that contribute to humoral immune responses. IL-4 appears to be differentially regulated in Th2 and Tfh cells^12^,^24^; however, the underlying molecular mechanisms of IL-4 expression in Tfh cells remain largely unknown. Our present study identifies a novel role of the transcription factor Batf in regulating IL-4 expression in Tfh cells. Batf deficiency leads to decreased IL-4 production, selectively in Tfh cells and subsequent reduction in their pro-allergic function. Batf–IRF4 complex along with transcription factors Stat3 and Stat6 bind to the CNS2 region of IL-4 locus and promote IL-4 production in Tfh cells.

Our *in vivo* results showed a T-cell-intrinsic defect in IL-4 production along with other effector Th2-related cytokines IL-5 and IL-13 in *Batf* KO mice. However, contrary to our expectations, analysis of *in vitro*-differentiated Th2 cells from *Batf* KO mice did not reveal any defect in the expression of the quintessential Th2 cytokine IL-4, while expression of IL-5 and IL-13 cytokines and the Th2 master transcription factor Gata3 were compromised (Supplementary Fig. 2) indicating the role of Batf in the regulation of selective Th2 factors rather than in global Th2 programming. In fact, Batf binds to the Gata3 locus indicating its role in the regulation of Gata3 expression and consequently in the production of Gata3-dependent cytokines IL-5 and IL-13. IL-4 is known to be extensively regulated by the combinatorial action of various transcription factors, *cis*-regulatory elements and epigenetic mechanisms in Th2 cells^4^,^5^,^22^. Although IL-4 has been widely recognized as a canonical marker for Th2-polarized CD4^+^ T cells^5^, Tfh is an alternative source of IL-4 (refs 2, 3, 24). Since deletion of Batf in T cells did not result in IL-4 defect in Th2 cells, Batf could contribute to IL-4 expression in Tfh cells. Interestingly, our *in vivo* studies revealed a novel non-redundant role of Batf in the regulation of IL-4 expression in Tfh cells compared with nTfh cells.

The CNS2 region, a 3′ enhancer in the IL-4 locus, is essential for IL-4 production by both Th2 and Tfh cells. However, although IL-4 expression in Tfh cells is dependent on CNS2, effector Th2 cells are far less dependent on this locus probably due to the redundancy of various DNA elements and transcription factors required to govern IL-4 expression in Th2 cells. We also observed increased binding of Batf to the CNS2 region of the IL-4 gene compared with IL-4 promoter region in Tfh cells (Fig. 3a). Although Batf lacks the transactivation domain, the transactivation observed at the CNS2 locus on Batf overexpression alone could be due to its interaction with endogenous factors (Fig. 3e). Batf is known to complex with AP-1/Jun family and cooperate with IRF4 to bind to AP-1-IRF4 composite elements in Th2, Th9 and Th17 cells^14^,^16^,^17^, however, whether the Batf-AP-1/IRF4 complexes exist in the Tfh subset and regulate its effector function have to be determined. Apart from enhanced IRF4 binding to the CNS2 region in Tfh cells (Fig. 3c), our immunoprecipitation experiments detected a physical interaction between Batf and IRF4 in *in vivo*-generated Tfh cells (Fig. 3f; Supplementary Fig. 7). Functionally, formation of Batf–IRF4 complex leads to transactivation of the CNS2 region in the IL-4 locus (Fig. 3e) indicating the positive cooperative role of Batf and IRF4 in IL-4 transcription in Tfh cells. In addition, blocking either Stat3 or Stat6 decreases the Batf–IRF4 function towards IL-4 induction in Tfh cells (Fig. 4g) suggesting the dependency of Batf–IRF4 complex on both Stat3 and Stat6. Whether Stat3 and Stat6 act alone or in a complex with Batf–IRF4 and therefore contribute to IL-4 expression in Tfh cells awaits further extensive investigation.

Apart from Batf and IRF4, we identified the positive effects of the transcription factor c-Maf in the regulation of IL-4 in Tfh cells. c-Maf is known to be a target of Batf in Tfh cells, which is required for regulation of IL-21 expression and consequently for Tfh expansion^25^. c-Maf is also known to control IL-4 expression in cooperation with IRF4 and NFAT in Th2 cells^26^. In our study, we also observed increased binding of c-Maf to the CNS2 locus in Tfh cells as well as c-Maf-induced transactivation of the CNS2 region indicating that c-Maf aids in IL-4 transcription in Tfh cells (Supplementary Fig. 5c,d). Since Batf is essential for c-Maf expression in Tfh cells, Batf-to-c-Maf signalling could additionally contribute to the decreased IL-4 expression in Tfh cells.

**Figure 7:**
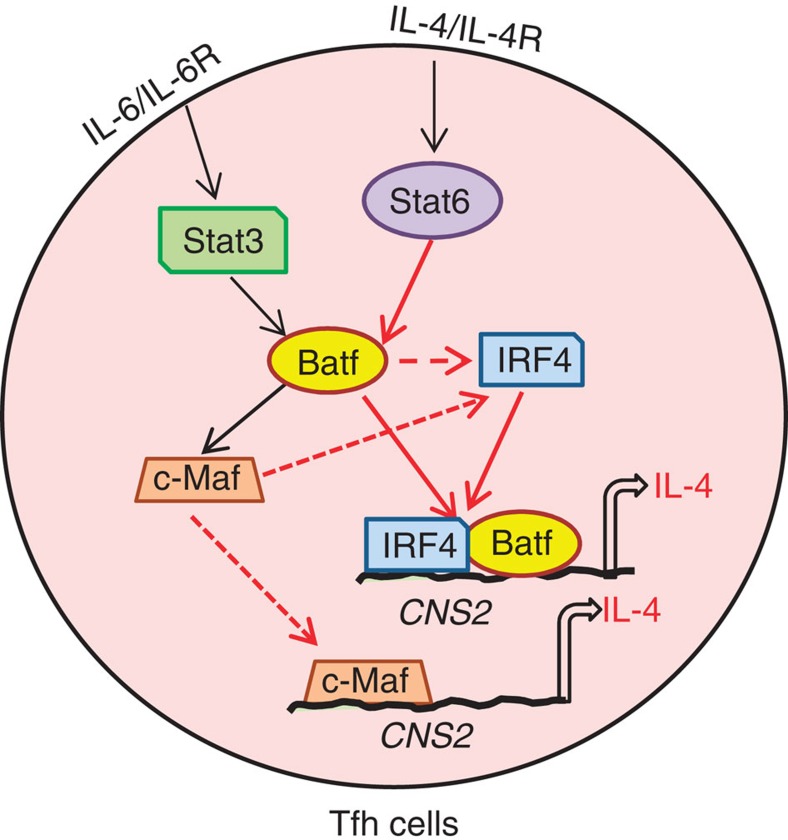
Schematic representation of Batf-mediated IL-4 regulation in Tfh cells. Batf expression in Tfh cells is regulated by IL-6/Stat3 and the IL-4/Stat6 pathway. Batf controls IRF4 expression and also partners with IRF4 to bind to the CNS2 region of IL-4 locus thus activating IL-4 transcription. c-Maf, a target gene of Batf also controls the expression of IRF4 and IL-4. Batf thus plays an important role in generation of IL-4-producing Tfh cells.

Our analysis of different Th subsets showed that Batf is highly expressed in Tfh and Th2 cells suggesting that both subsets could share the similar mechanism for Batf expression. Similar to Th2 cells, we found that the IL-4/Stat6 pathway is important for Batf expression and hence for IL-4 production in Tfh cells (Fig. 6). However, the requirement of IL-4–Stat6–Batf axis could be dispensable since it has been reported that IL-4-producing Tfh cells could be developed even on downregulation of the IL-4 receptor and in the absence of IL-4 (refs 2, 30). Batf expression in Tfh cells is known to be also induced by IL-6 signalling via the transcription factor Stat3 (ref. 13). Thus, Batf induction in Tfh cells by IL-6 or IL-4 through Stat3 or Stat6, respectively, helps in the generation of IL-4-expressing Tfh cells (Fig. 7). However, the exact kinetics of IL-4/Stat6 and IL-6/Stat3 contribution in Batf-mediated IL-4 expression in Tfh cells needs further extensive investigation.

In summary, our results identify a previously undetermined role of Batf in controlling IL-4 expression in Tfh cells. Batf in the complex with IRF4 and along with Stat3 and Stat6 binds to the CNS2 region in the IL-4 gene locus and facilitates IL-4 transcription in Tfh cells. Since IL-4 has been implicated in the pathogenesis of allergic diseases, IL-4 expression in Tfh cells contributes to their pro-allergic function. Specifically, deletion of Batf in Tfh cells protects mice from induction of allergic asthma; Batf may be targeted in treating allergic inflammatory diseases.

## Methods

### Mice and cell line

*Batf* KO, *TCR-β* KO, *Rag1* KO, *Stat6* KO and C57BL/6J mice were purchased from Jackson laboratory. *Stat3*^*f/f*^ mice^31^ were bred with *CD4-Cre* mice^32^. Six- to eight-week-old age- and sex-matched mice were used for all experiments. Mice were housed in a specific pathogen-free animal facility at MD Anderson Cancer Center, and all animal experiments were performed according to protocols approved by Institutional Animal Care and Use Committee. The EL-4 cell line was obtained and cultured according to instructions from American type culture collection (ATCC).

### T-cell differentiation

Fluorescence-activated cell sorting (FACS)-sorted naive CD4^+^CD25^−^CD62L^hi^CD44^lo^ T cells from splenocytes and lymph node cells of WT and *Batf* KO mice were activated with plate-bound anti-CD3 and anti-CD28 (BD Pharmingen, 2 μg ml^−1^) and polarized under various Th conditions as described previously^33^. The culture conditions are as follows: Th0 (50 U ml^−1^ hIL-2, PeproTech); Th1 (10 ng ml^−1^ IL-12, PeproTech), 50 U ml^−1^ hIL-2, 10 μg ml^−1^ anti-IL-4 (Bioexcel); Th2 (10 ng ml^−1^ IL-4, PeproTech), 50 U ml^−1^ hIL-2 and 10 μg ml^−1^, anti-IFN-γ (Bioexcel); Th17 (20 ng ml^−1^ IL-6, PeproTech), 2 ng ml^−1^ TGF-β (PeproTech), 10 μg ml^−1^ anti-IFN-γ and 10 μg ml^−1^ anti-IL-4); and iTreg (2 ng ml^−1^ TGF-β, 10 μg ml^−1^, anti-IFN-γ and 10 μg ml^−1^ anti-IL-4). Four days after culture, differentiated cells were restimulated with plate-bound anti-CD3 for 4 h and analysed.

### Quantitative real-time PCR

Total RNA was extracted from either *in vitro*-cultured Th cells or *in vivo*-generated Tfh and nTfh cells or lung tissues using TriZol reagent (Invitrogen) according to manufacturer's instructions. Oligonucleotide (dT) and Moloney murine leukemia virus (MMLV) reverse transcriptase (Invitrogen) were used to synthesize cDNA. Gene expression of beta-actin (*Actb*), *Il4*, *Il5*, *Il13*, *Gata3* (ref. 34), *Batf*, *c-Maf*^15^ and *Irf4* (ref. 15) was examined using the iQ SYBR Green real-time PCR kit (Bio-Rad Laboratories, Inc.). The data were normalized to the reference gene beta-actin. The primer pair's sequences are listed in Table 1.

### Retroviral transduction

Retroviral transduction was performed as described previously with minor modifications^35^. Briefly, naive CD4^+^CD25^−^CD62L^hi^CD44^lo^ T cells from C57BL/6J (WT) and *Stat6* KO mice were sorted and activated with plate-bound anti-CD3 and anti-CD28 (2 μg ml^−1^, BD Pharmingen) under neutral condition. Twenty-four hours after activation, cells were infected by retroviruses expressing Stat6 or control empty vector (containing only internal ribosomal entry site-green fluorescent protein (IRES–GFP)). Three days after infection, FACS-sorted GFP^+^ cells were restimulated with anti-CD3 for 4 h and *Batf* mRNA expression was analysed by qRT–PCR.

### Chromatin immunoprecipitation assay

Cells were fixed with 1% formaldehyde for 10 min at 37 °C followed by ChIP with the Millipore ChIP kit. Briefly, chromatin was seared (500 bp–100 kb) and immunoprecipitated using the antibodies (Abs) to the following: AcH3, H3k4me3 and rabbit IgG (Millipore); Stat3, Stat6, Batf and IRF4 (Santa Cruz Biotechnology). Complexes of DNA and antibodies were precipitated using protein G agarose (Millipore) and then reverse crosslinked at 65 °C for 4 h. Following reversal of cross-links, the presence of selected DNA sequences was assessed by qRT–PCR at the 5′ region of various gene loci using the primer sequences listed in Table 1. The data from each replicate are normalized to the input control and represented as fold enrichment to control antibody (rabbit IgG).

### Coimmunoprecipitation assay

Briefly, total Tfh cell lysates were prepared by lysing cells in Triton lysis buffer supplemented with protease inhibitor cocktail (Roche) and phosphatase inhibitors (10 mM NaF and 1 mM Na_3_VO_4_). IRF4 protein in the cell lysates was immunoprecipitated using anti-IRF4 antibody (Santa Cruz Biotechnology, 5 μg per sample) followed by detection of Batf by immunoblotting with anti-Batf antibody (Santa Cruz Biotechnology, 1:500 dilution).

### Ovalbumin immunization

Male WT (C57BL/6J), *Batf* KO, *Stat3* KO and *Stat6* KO mice (6–8 weeks old; *n*=5 per group) were intraperitoneally (i.p.) immunized with chicken Ova protein (1.0 mg ml^−1^) emulsified in Alum. Seven days after immunization, spleen cells were stimulated with 100 μg ml^−1^ Ova protein for 72 h and stained with Pacific blue-labelled Sytox, PerCP-labelled anti-CD4 mAb, FITC-labelled anti-CD44 mAb and biotinylated anti-CXCR5 mAb followed by allophycocyanin (APC)-labelled streptavidin (BD Biosciences). CD4^+^CD44^hi^CXCR5^hi^PD1^hi^ (Tfh) and CD4^+^CD44^hi^CXCR5^−^ (nTfh) cells were then FACS sorted and re-sorted to confirm the purity of the populations and later analysed by qRT–PCR and ELISA. Spleen cells were also cultured for 72 h without or with different concentrations of Ova protein and effector cytokines were analysed 72 h later by ELISA (PharMingen).

### Adoptive transfer and mixed bone marrow chimera studies

Adoptive transfer and bone marrow chimera experiments were done as described previously^35^. Briefly, FACS-sorted naive CD4^+^ T cells from male WT or *Batf* KO mice (6–8 weeks old) were intravenously transferred into male *TCR-β* KO mice (6–8 weeks old, 10 × 10^6^ cells per mouse; *n*=4 per group) followed by Ova in Alum immunization. Seven days post immunization, spleen cells from the recipient mice were stimulated for 3 days with Ova and WT and *Batf* KO CD4^+^CD44^hi^CXCR5^hi^PD1^hi^ (Tfh) and CD4^+^CD44^hi^CXCR5^−^ (nTfh) cells were sorted as described above and levels of effector cytokines were analysed by qRT–PCR and ELISA. The IgG levels in the serum of the recipient mice were also analysed by ELISA.

Naïve CD4^+^ T cells from OTII and *Batf* KO OTII mice (6–8 weeks old) were FACS sorted and transferred into B6.SJL (CD45.1^+^) mice (10 × 10^6^ cells per mouse, *n*=5 per group) followed by immunization with Ova in Alum. Seven days after immunization Tfh and nTfh cells were sorted from the spleen of these mice as described above for further analysis.

For bone marrow chimera experiments, bone marrow cells from male B6.SJL (CD45.1^+^) or *Batf* KO (CD45.2^+^) mice (6–8 weeks old) were mixed at 1:1 ratio and transferred into irradiated male *Rag1*^−/−^ mice (6–8 weeks old, 10 × 10^6^ cells per mouse and 750 rad). Eight weeks later, the reconstituted mice were subjected to immunization with Ova protein in Alum and analysed as described above.

### Asthma induction

Allergic asthma was induced and analysed as described previously^36^. Briefly, male WT (C57BL/6J) and *Batf* KO mice (6–8 weeks old, *n*=5 per group) were i.p. immunized twice at 2 weeks interval with 0.2 ml saline containing 100 μg Ova in Alum. Mice were intranasally sensitized with Ova on day 14 and rechallenged intranasally three more times at days 26, 27 and 28. Twenty-four hours after the last challenge mice were killed and the bronchoalveolar lavage fluid (BALF), serum, lungs, spleens and the lung lymph nodes were collected. BALF was analysed for cellular composition using the Differential Quik Stain kit (Modified Giemsa). The lung was used for RNA extraction and spleen- and lung-draining mediastinal lymph node cells were further cultured with Ova for 72 h and supernatants were analysed for cytokine expression by ELISA.

For the adoptive transfer model of asthma, FACS-sorted WT (CD45.1^+^) and *Batf* KO (CD45.2^+^) Tfh cells from the lung and lung lymph nodes of bone marrow chimera mice subjected to asthma were intravenously transferred into male C57BL/6J (CD45.2^+^) or B6.SJL (CD45.1^+^) mice (6–8 weeks old), respectively (4 × 10^5^ cells per mouse). The recipient mice were challenged intranasally with Ova for 5 days post transfer and analysed as indicated above 24 h after the last challenge.

To confirm antigen specificity, WT OTII and *Batf* KO OTII Tfh cells were sorted from male asthmatic mice (6–8 weeks old), which received either naive WT OTII or *Batf* KO OTII cells. Sorted cells were transferred into male B6.SJL (CD45.1^+^) mice (6–8 weeks old, 10^5^ cells per mouse) followed by intranasal challenge with Ova for 5 days post transfer. Twenty-four hours after the last challenge the BALF, spleens and serum of the immunized mice were used for further analysis.

### Statistical analysis

All results are displayed as mean values±s.e.m. Comparison between two different groups were done using unpaired two-tailed Student's *t*-test. Statistical analyses were performed with GraphPad Prism software (version 6.0, GraphPad software, San Diego, CA) and *P* values <0.05 were considered significant. All experiments were performed at least thrice with comparable results.

## Additional information

**How to cite this article:** Sahoo, A. *et al*. Batf is important for IL-4 expression in T follicular helper cells. *Nat. Commun.* 6:7997 doi: 10.1038/ncomms8997 (2015).

## Supplementary Material

Supplementary Information Supplementary Figures 1-7

## Figures and Tables

**Table 1:** List of mRNA and ChIP qRT–PCR primer sequences.

**Real-time qRT–PCR primers**		
**Gene**	**Forward primer**	**Reverse primer**
*Actb*	5′-TGGAATCCTGTGGCATCCATGAAAC-3′	5′-TAAAACGCAGCTCAGTAACAGTCCG-3′
*Il4*	5′-AGATCACGGCATTTTGAACG-3′	5′-TTTGGCACATCCATCTCCG-3′
*Il5*	5′-CGCTCACCGAGCTCTGTTG-3′	5′-CCAATGCATAGCTGGTGATTTTT-3′
*Il13*	5′-GCTTATTGAGGAGCTGAGCAACA-3′	5′-GGCCAGGTCCACACTCCATA-3′
*Gata3*	5′-AGGGACATCCTGCGCGAACTGT-3′	5′-CATCTTCCGGTTTCGGGTCTGG-3′
*c-maf*	5'-AGCAGTTGGTGACCATGTCG-3'	5'-TGGAGATCTCCTGCTTGAGG-3'
*Batf*	5′-GTTCTGTTTCTCCAGGTCC-3′	5′-GAAGAATCGCATCGCTGC-3′
*Irf4*	5'-GCCCAACAAGCTAGAAAG-3'	5'-TCTCTGAGGGTCTGGAAACT-3'

**ChIP qRT–PCR primers**		
**5**′ **region of gene**	**Forward primer**	**Reverse primer**
Batf	5′-CCTCAGGCTTGTCGCTGACT-3′	5′-CACATGGGCGGAAACGTCAAC-3′
CNS2	5′-AATCTTAAAAGTGGGGAAGGGG-3′	5′-GATCTAATGGTTGCCTCCTTA-3′
IL-4	5′-ACTCATTTTCCCTTGGTTTCAGC-3′	5′-GATTTTTGTCGCATCCGTGG-3′
IL-5	5′-AGATGCAGGCGTGCGATGTT-3′	5′-TCTCAATCTGGAGGACAGGGC-3′
Gata3	5′-CTTGGCGTCAGCAGCTTTCT-3′	5′-CCAGCATCTCTTCCAGCTCAC-3′
IRF4	5′-TGAAGCTGTTTGGGTGGGAG-3′	5′-GGAATCACGCTCTGGAGCC-3′
