# Supplementary material for: Batf is important for IL-4 expression in T follicular helper cells
Source: Nat Commun. 2015 Aug 17;6:7997. doi: 10.1038/ncomms8997 (PMC4557271; doi:10.1038/ncomms8997)
Supplement: Supplementary Information — Supplementary Figures 1-7 [file ncomms8997-s1.pdf]

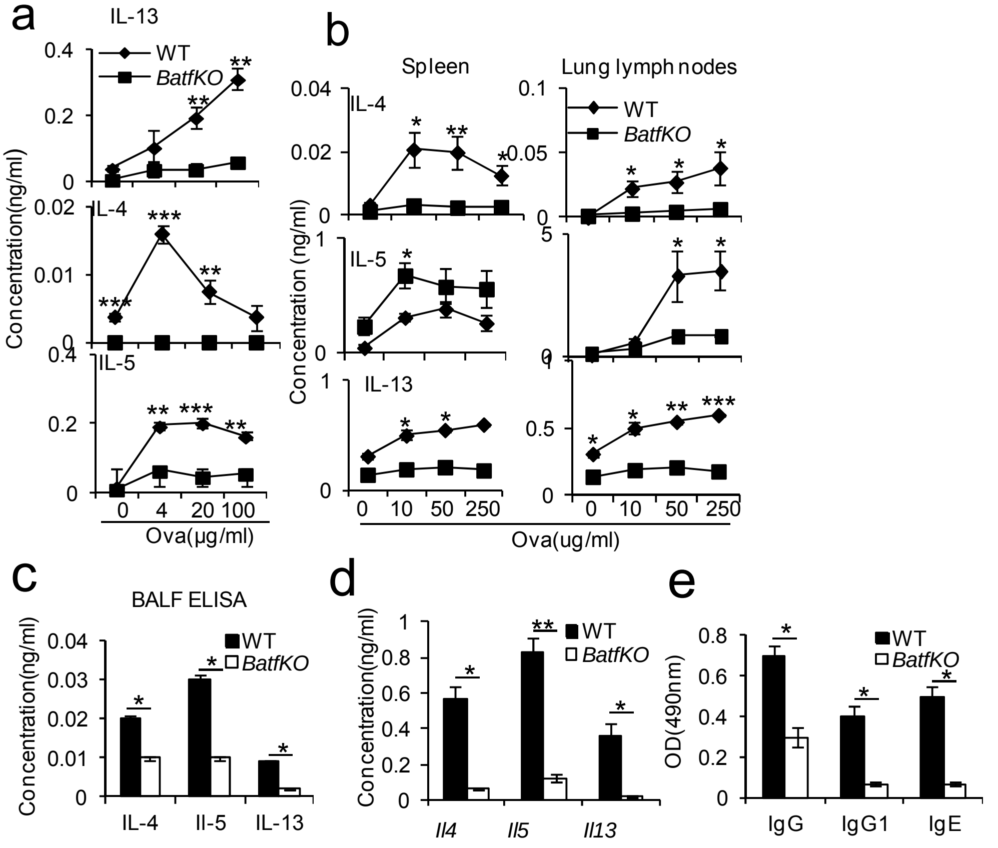

Supplementary Figure 1. Batf controls Th2 responses *in vivo*. (**a**) Male WT and *Batf* KO mice (6-8 weeks old, n=5 per group) were i.p. sensitized with Ova in alum. One week post immunization, splenocytes were restimulated with different concentrations of Ova for 72 hrs and Th2 cytokines were measured by ELISA. (**b-c**) Male WT and *Batf* KO mice (6-8 weeks old, n=5 per group) were i.p. sensitized with Ova in alum twice at 2 weeks interval, followed by intranasal challenge with Ova for 3 days. (**b**) Splenocytes and lung lymph node cells from WT and *Batf* KO mice were restimulated with different concentrations Ova for 72 hrs and Th2 cytokines were measured by ELISA. (**c**) Protein levels of Th2 cytokines in BALF were analyzed by ELISA. (**d-e**) FACS sorted naive $CD4^+$ T cells from male WT and *Batf* KO mice were intravenously transferred into male *TCR-β* KO mice (6-8weeks old, 10 million cells per mouse, n=4 per group) followed by intraperitoneal injection with Ova in alum. Seven days later recipient *TCR-β* KO mice were sacrificed for analysis. (**d**) Splenocytes from recipient mice were restimulated with different concentrations Ova for 72 hrs and Th2 cytokines were measured by ELISA. **(e)** Immunoglobulin levels in the serum of recipient *TCR-β* KO mice were analyzed by ELISA. The results shown are mean ± SEM. P values: *<0.05, **<0.01, ***0.001. Student's t-test was performed to detect between-group differences. The data are representative of at least three independent experiments with consistent results.

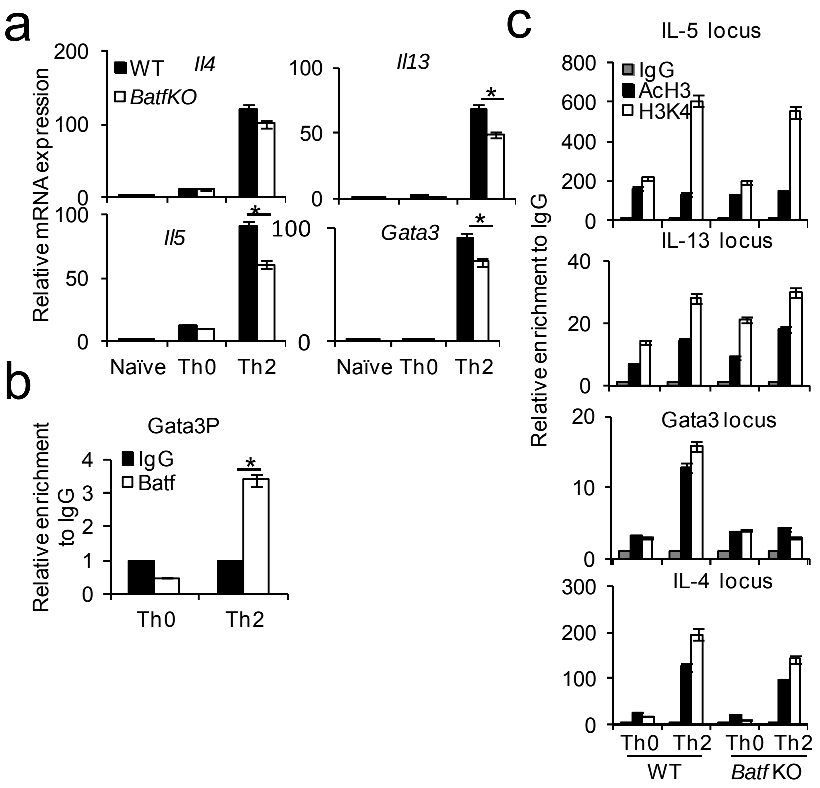

Supplementary Figure 2. Expression of T helper 2 cytokines in *Batf* KO CD4$^+$ T cells. Naïve CD4$^+$ T cells from male WT and *Batf* KO mice (6-8weeks old) were isolated and cultured for 4 days under Th0 and Th2 polarizing cell conditions. (**a**) mRNA levels of the indicated genes were analyzed by qRT-PCR. The data shown were normalized by the expression of a reference gene *Actb*. (**b**) Batf binding to the *Gata3* promoter (Gata3P) locus in Th2 cells was analyzed by chromatin immunoprecipitation (ChIP) assay. (**c**) ChIP analysis of active histone proteins, H3 acetylation (AcH3) and trimethyl histone H3 lysine 4 (H3k4) at the indicated loci. The graphs shown in (**b**) and (**c**) represent fold enrichment of the indicated proteins to control antibody (rabbit IgG) at the designated locus. The data from each replicate were normalized to the input control. The results shown are mean ± SEM. P values: *<0.05. Student's t-test was performed to detect between-group differences. The data are representative of at least three independent experiments with consistent results.

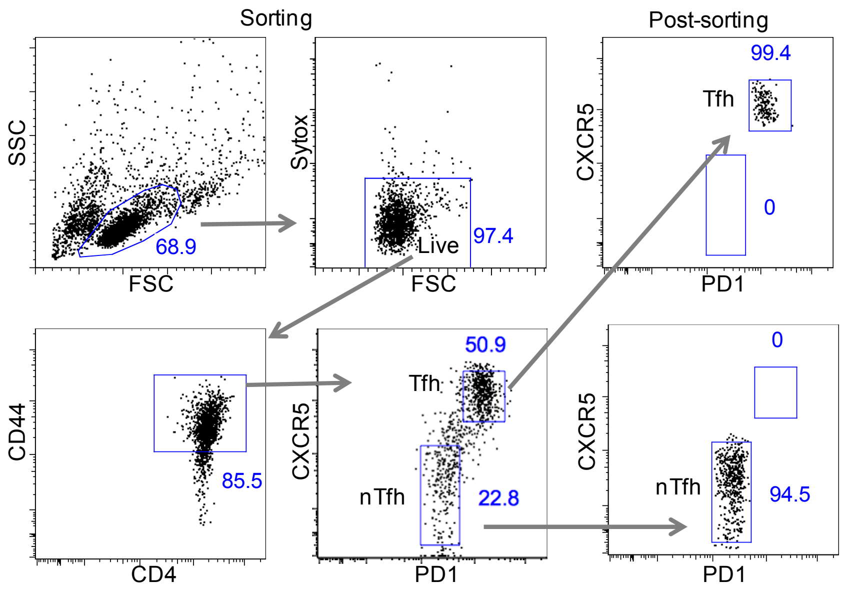

Supplementary Figure 3. Gating strategy of CD4^{+}CD44^{hi}CXCR5^{hi}PD1^{hi} (Tfh) and CD4^{+}CD44^{hi}CXCR5^{-} (nTfh) cells. Male WT mice (6-8 weeks old, n=5) were injected i.p. with 0.2 ml saline containing 100 µg Ova in Alum. Seven days later, splenocytes from immunized mice were isolated and stimulated with ova (100ug/ml) for 72 hrs *ex vivo* and stained with Pacific blue-labeled Sytox, PerCP-labeled anti-CD4 mAb, FITC-labeled anti-CD44 mAb, PE labeled anti-PD1 mAb and biotinylated anti-CXCR5 mAb, followed by APC-labeled streptavidin (BD Biosciences). CD4^{+}CD44^{hi}CXCR5^{hi}PD1^{hi} (Tfh, 99.4% purity) and CD4^{+}CD44^{hi}CXCR5^{-} (nTfh, 94.5% purity) cells were then sorted as depicted for further analysis.

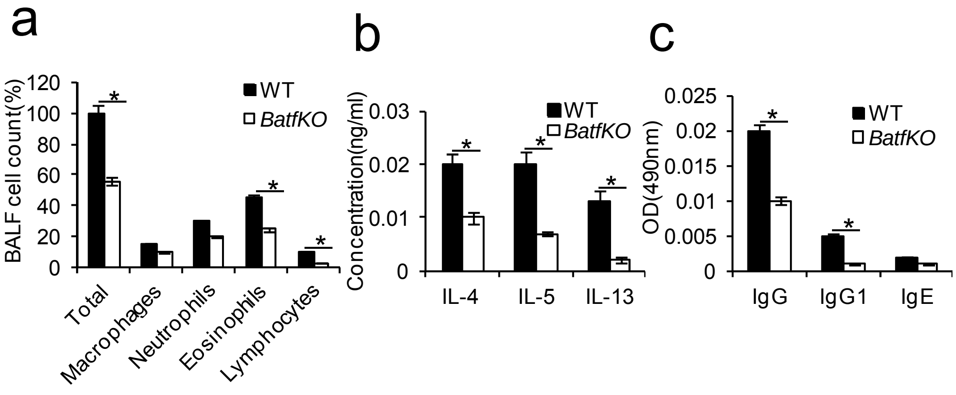

Supplementary Figure 4. Batf controls Th2 responses *in vivo.* Naïve WT OT II and *Batf*KO OT II cells were transferred into male B6.SJL (CD45.1$^+$) mice (6-8 weeks old, 10 million cell per mouse, n=4 per group) and recipient mice were immunized twice intraperitoneally at 2 weeks interval with 0.2 ml saline containing 100 μg Ova in Alum. On day 14, mice were intranasally challenged with Ova followed by three more challenges at day 26, 27 and 28. 24 hours post the last challenge, mice were sacrificed and FACS sorted antigen-specific donor WT and *Batf* KO CD45.2$^+$CD4$^+$CD44^{hi}CXCR5^{hi}PD1^{hi} Tfh cells from lung and lung lymph nodes of the above mice were intravenously transferred into male B6.SJL (CD45.1$^+$) mice (6-8 weeks old, n=4 per group, 10^5 cells/mouse). The recipient mice were challenged intranasally with Ova for 5 days post transfer and analyzed 24 hours after the last challenge. (**a**) Percentage of total infiltrated cells, macrophages, neutrophils, eosinophils and lymphocytes in recipient mice. (**b**) ELISA of cytokines IL-4, IL-5 and IL-13 in Ova restimulated splenocytes. (**c**) ELISA of immunoglobulins in the sera of recipient mice. The results shown are mean ± SEM. P values: *<0.05. Student's t-test was performed to detect between-group differences. The data are representative of at least three independent experiments with consistent results.

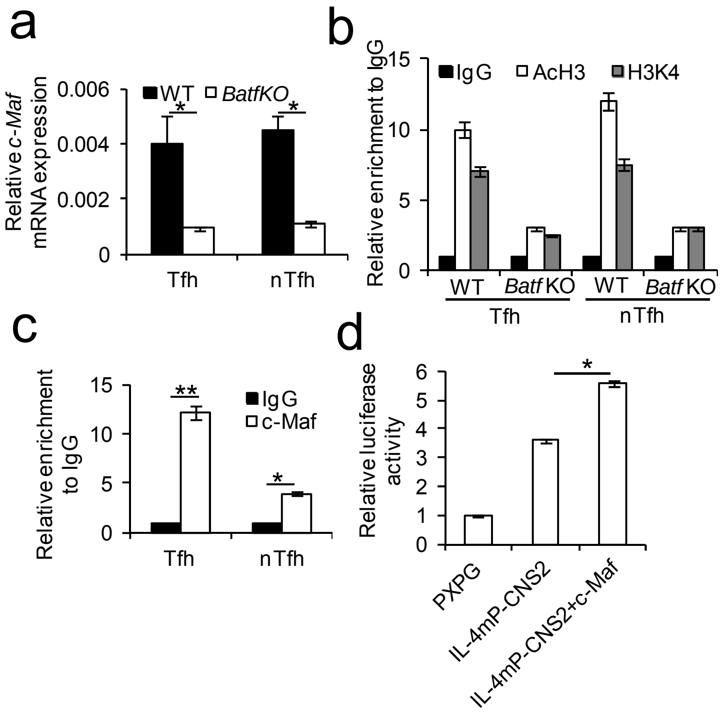

Supplementary Figure 5. **Batf-c-Maf axis in regulation of IL-4 expression in Tfh cells.** Male WT and *Batf* KO mice (6-8 weeks old) were injected i.p with Ova in alum. Seven days later, $CD4^{+}CD44^{hi}CXCR5^{hi}PD1^{hi}$ (Tfh) and $CD4^{+}CD44^{hi}CXCR5^{-}$ (nTfh) cells were sorted from the spleen of immunized mice. (**a**) *c-Maf* mRNA levels in Tfh and nTfh cells were analyzed by qRT-PCR. The data shown were normalized by the expression of a reference gene *Actb*. (**b**) ChIP analysis of active histone proteins (AcH3 and H3k4) at the *c-Maf* gene loci in Tfh and nTfh cells. (**c**) c-Maf binding to the CNS2 region of IL-4 locus in Tfh and nTfh cells was analyzed by ChIP assay. The graphs shown in (**b**) and (**c**) represent fold enrichment of the indicated proteins to control antibody (rabbit IgG) at the designated locus. The data from each replicate were normalized to the input control. (**d**) Luciferase assay in EL-4 cells transfected with IL-4mP-CNS2 luciferase vector along with c-Maf expression plasmid. Relative luciferase units are expressed as a fold difference to the control (pGL3) value. The results shown are mean ± SEM. P values: *<0.05. Student's t-test was performed to detect between-group differences. The data are representative of at least three independent experiments with consistent results.

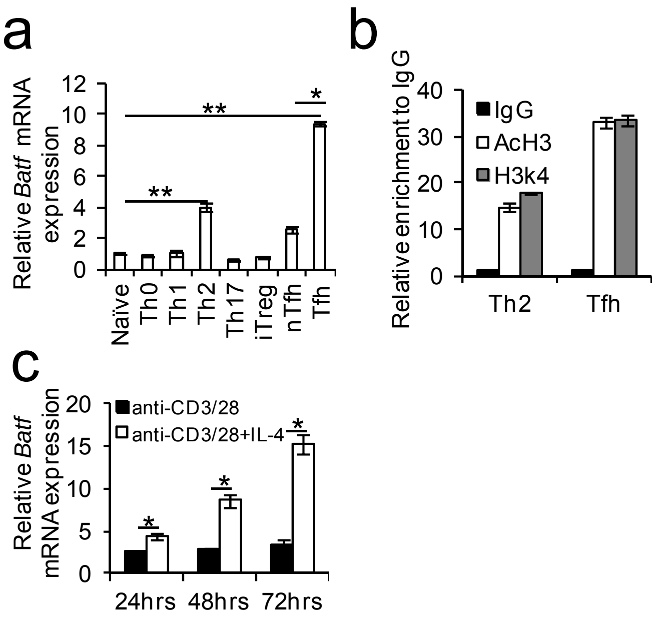

Supplementary Fig. 6. Expression of *Batf* in T helper cells. (**a**) Naïve T cells were cultured for 4 days under various polarizing conditions. Male C57BL/6J mice (6-8 weeks old) were immunized i.p with Ova in alum and $CD4^{+}CD44^{hi}PD1^{hi}CXCR5^{+}$ (Tfh) and $CD4^{+}CD44^{hi}CXCR5^{-}$ (nTfh) cells were sorted one week post immunization. *Batf* mRNA expression was analyzed in all subsets by qRT-PCR. The data shown were normalized by the expression of a reference gene *Actb*. (**b**) ChIP analysis of indicated histone proteins at Batf promoter locus in Th2 and Tfh cells. The graphs represent fold enrichment of the indicated proteins to control antibody (rabbit IgG) at the designated locus. The data from each replicate were normalized to the input control. (**c**) Naïve $CD4^{+}$ T cells were cultured under indicated conditions and *Batf* mRNA expression was analyzed at different time points by qRT-PCR. The data shown were normalized by the expression of a reference gene *Actb*. The results shown are mean ± SEM. P values: *<0.05, **<0.01. Student's t-test was performed to detect between-group differences. The data are representative of at least three independent experiments with consistent results.

Fig. 3f

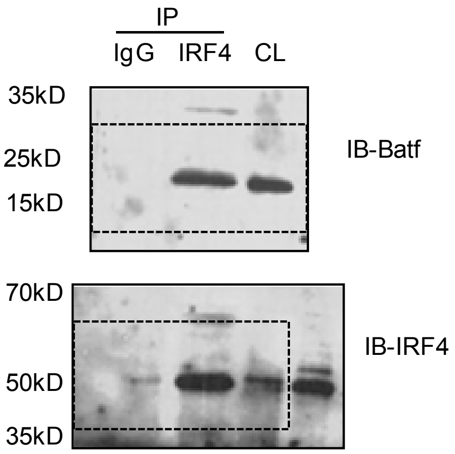

Supplementary Figure 7. Uncropped gel images.
